# Supplementary material for: Epidemiology of injured patients in rural Uganda: A prospective trauma registry’s first 1000 days
Source: PLoS One. 2021 Jan 22;16(1):e0245779. doi: 10.1371/journal.pone.0245779 (PMC7822551; doi:10.1371/journal.pone.0245779)
Supplement: S1 Appendix — (PDF) [file pone.0245779.s002.pdf]

SRRH TRAUMATIC INJURY FORM v.1i

Questions? Call Mary G. 0704412718

Patient ***SURNAME, Other*** ______________________

AGE ____ ***SEX*** M / F ***DISTRICT*** __________ ***SUB-COUNTY*** __________ ***VILLAGE*** __________

INJURED on __ / __ / __ (day month year) at __ : __ AM PM

ARRIVED on __ / __ / __ (day month year) at __ : __ AM PM

OCCUPATION
- O Peasant farmer
- O Student/pupil
- O None
- O Other: __________

MODE OF ARRIVAL
- O Motorcycle/taxi
- O By foot
- O Private car or bicycle
- O Ambulance or police

REFERRED?
- O No
- O Yes, from: __________

INJURY MECHANISM

- O Fall (☐ *from height* ☐ *ground level*)
- O Burn
- O Gunshot
- O Blunt force
- O Animal bite
- O Other: __________
- O Stab/cut
- O Poisoning

O Road Traffic Accident (FILL ALL):

Patient using?	*Patient role?*	*Helmet?*
☐ Vehicle	☐ Driver / Rider	☐ Yes
☐ Motorcycle	☐ Passenger	☐ No
☐ Bicycle	☐ Pedestrian	☐ N/A

ADDITIONAL HISTORY OF PRESENT COMPLAINT

....

....

SETTING
- O Home
- O School
- O Work
- O Road/street
- O Other: ________

INTENT
- O Unintentional
- O Intentional, assault
- O Intentional, self-harm
- O Mob justice

RECENT ALCOHOL/DRUGS?
- O No
- O Confirmed/suspected

HIV STATUS
- O TR/TRK
- O TRR/TRRK

☐ PERFORM PRIMARY SURVEY OF PT'S: **A**IRWAY | **B**REATHING | **C**IRCULATION | **D**ISABILITY | **E**XPOSURE

INITIAL SYSTOLIC BP
- O >89 mmHg
- O 50-89 mmHg
- O 1-49 mmHg
- O Undetectable
- O *BP CUFF UNAVAILABLE*

Systolic ---------- *Diastolic*

INITIAL PR
- O 60-100 bpm
- O >100 bpm
- O <60 bpm
- O Undetectable

INITIAL RESP. RATE
- O 10-29/minute
- O 30+/minute
- O <9/minute

NEURO STATUS
- O Alert
- O Responds to verbal stim.
- O Responds to painful stim.
- O Unresponsive

or GCS = ____

No. OF SERIOUS INJURIES
- O Zero
- O One
- O Multiple

"Serious injury": requires hospital admission

INJURED BODY AREA(S)
☐ Head/Neck ☐ Face ☐ Chest ☐ Abdomen/Pelvis/Perineum ☐ Back/Spinal Cord ☐ Upper Extremity ☐ Lower Extremity

DIAGNOSIS
- ☐ Head injury
- ☐ Thoracic injury
- ☐ Burn
- ☐ Laceration/bite
- ☐ Spinal cord injury
- ☐ Bruise/abrasion (*Soft tissue injury*)
- ☐ Abdominal injury
- ☐ Fracture (☐ *CLOSED* ☐ *OPEN*)
- ☐ Sprain/dislocation
- ☐ Other __________

ADD. DIAG. DETAILS

....

....

INITIAL DECISION
- ☐ Treat with __________ & send home
- ☐ Take to theatre
- ☐ N/A (*died*)
- ☐ Admit to hospital ward No. ___
- ☐ Send to __________

First seen by

name __________

position __________

location __________

on __ / __ / __ (*day month year*) at __ : __ *AM PM*

If not same as above, form completed by __________

If admitted, fill below:

IMAGING DURING ADMISSION: ☐ X-Ray ☐ Ultrasound ☐ CT

GIVEN BLOOD? Yes, ____ units

MEDICATION(S) DURING ADMISSION: ....

If no medications administered, write "NONE"

PROCEDURE(S) DURING ADMISSION: ....

If no procedures performed, write "NONE"

COMPLICATION(S) DURING ADMISSION: ....

For example: DVT/PE, pneumonia, urinary tract infection, surgical site infection, unplanned reoperation, sepsis, etc.

WAS A DIAGNOSTIC STUDY AND/OR TREATMENT RECOMMENDED BUT NOT PERFORMED/RECEIVED?

If yes, what and why not? ....

INPATIENT DISPOSITION
- ☐ Discharged
- ☐ Transferred, to __________ for __________
- ☐ Escaped/ran away
- ☐ Left against medical advice
- ☐ Died (*EXPECTED DEATH* ☐ *UNEXPECTED DEATH* ☐)

on __ / __ / __ (*day month year*) at __ : __ *AM PM*

DISABILITY AT DISCHARGE
- ☐ Mild — *Able to return to work/school*
- ☐ Moderate — *Independent at home but unable to return to work/school*
- ☐ Severe — *Dependent on others at home*
