# Supplementary material for: Epidemiology of injured patients in rural Uganda: A prospective trauma registry’s first 1000 days
Source: PLoS One. 2021 Jan 22;16(1):e0245779. doi: 10.1371/journal.pone.0245779 (PMC7822551; doi:10.1371/journal.pone.0245779)
Supplement: Attachment — Submitted filename: Response to Reviewers.docx [file pone.0245779.s003.docx]

**RESPONSE TO REVIEWERS**

The original comments by Reviewer 1 and Reviewer 2 are listed below, followed by our response to each comment:

Reviewer 1:

1. *As per the recent SABRE guidelines I suggest where you refer to GENDER this is changed to SEX (the former is self-determined and the latter genetically fixed)*

This has been corrected in each instance.

2. *Some minor expansion of the discussion to include not only the other Ugandan reports on injury burden, but also the other countries around Uganda would be useful (recent work from Botswana and Tanzania published in WJS or AfJEM and from Malawi in various journals would be useful for comparison, not to mention the numerous papers detailing a very different injury pattern from South Africa*

Studies describing findings from other sub-Saharan African trauma registries were added to the discussion in order to better contextualize our work.

3. *A discussion around the NSOAP recently completed for Uganda would be useful to contextualize the paper*

We were unable to discover much about the current status of Uganda’s NSOAP in the literature, nor are our local collaborators involved in its development, but this will undoubtedly be a landmark step in the advancement of care for surgical patients in Uganda.

4. *A clear conclusion that is not stated strongly enough as a recommendation would be the establishment of a proper Emergency Department at the hospital.*

This is a valuable lesson learned from our registry implementation and has been better emphasized in our conclusion.

Reviewer 2:

1. *I understand the reason why the KTS was used, it makes comparison with other international studies difficult. Furthermore, this score mainly contains physiological parameters, parameters which are known to be missing when not collected miticuleously (in the present study 48% missing data). And when they are collected, these parameters are severely influenced by the pre-hospital setting (distance/time, treatment given/not given etc). Is additional information available for (at least a part of) these patients, such as ISS, NISS? The advantage of these scores based on anatomy is they can be collected in hindsight.*

Many trade-offs were made in the development of the registry’s instrument, in particular balancing the desire for detailed data collection while maintaining the feasibility of the project. The hospital’s workflow demands and lack of widespread clinical expertise prevented us from reliably establishing an Injury Severity Score for the patients in the registry, whether prospectively or retrospectively. Previously published work from our group has demonstrated the significant predictive value for hospital mortality of physiologic scoring systems such as the Kampala Trauma Score (KTS). We felt this approach would maximize practicality and allow for adequate comparisons with other trauma registries located in low- & middle-income settings. The inability to reliably record all of the elements making up the KTS is a multifactorial problem and represents a focus of ongoing quality improvement measures, in part driven by the trauma registry.

2. *A statement is made that the registry influences trauma outcome. Do the authors have data on this statement? Can this be further elaborated?*

In our discussion we have described in further detail the possible downstream effects of trauma registry implementation. Given the extensive education efforts associated with the establishment and maintenance of the registry, it is not inconceivable that clinical providers could have altered their behaviors to emphasize collection of vital signs, for example. While the Hawthorne effect may influence the practice of clinical care in this way, we would hesitate to say that it may affect patient outcomes.

3. *The number of patients included in the registry declines over time (each period, the number is cut in half). This might be one of the most important findings of this paper. Can there be reasons identified for this phenomenon? How can other centers learn from this experience?*

In our revised discussion we describe in further detail the varied factors contributing to the decline in registry patients over time. They encompass a range of barriers to success in global health, many of which have fatally afflicted trauma registries around the world. Although our data collection is not as robust as it once was, we have successfully adapted our approach to meet the challenge of sustainability, and we consider our currently operational database to serve as an example of the immense capabilities of a locally-run trauma registry.
